# Not water, sanitation and hygiene practice, but timing of stunting is associated with recovery from stunting at 24 months: results from a multi-country birth cohort study

**DOI:** 10.1017/S136898002000004X

**Published:** 2021-04

**Authors:** Subhasish Das, Shah Mohammad Fahim, Md Ashraful Alam, Mustafa Mahfuz, Pascal Bessong, Esto Mduma, Margaret Kosek, Sanjaya K Shrestha, Tahmeed Ahmed

**Affiliations:** 1 Nutrition and Clinical Services Division, International Centre for Diarrheal Disease Research (icddr,b), Bangladesh; 2 Faculty of Medicine and Life Sciences, University of Tampere, Tampere, Finland; 3 University of Venda, South Africa; 4 Haydom Global Health Institute, Haydom, Tanzania; 5 Division of Infectious Diseases & International Health, University of Virginia, Charlottesville, VA, USA; 6 Walter Reed/Armed Forces Research Institute of Medical Sciences (AFRIMS) Research Unit Nepal (WARUN), Kathmandu, Nepal

**Keywords:** Recovery from stunting, Water, sanitation and hygiene, Timing of stunting, Generalised linear mixed-effects models, Multi-country birth cohort study

## Abstract

**Objectives:**

To measure the role of water, sanitation and hygiene (WASH) practices on recovery from stunting and assess the role of timing of stunting on the reversal of this phenomenon

**Design:**

Data from the MAL-ED multi-country birth cohort study was used for the current analysis. Generalised linear mixed-effects models were used to estimate the probability of reversal of stunting with WASH practice and timing of stunting as the exposures of interest.

**Setting:**

Seven different countries across three continents.

**Participants:**

A total of 612 children <2 years of age.

**Results:**

We found that not WASH practice but timing of stunting had statistically significant association with recovery from stunting. In comparison with the children who were stunted at 6 months, children who were stunted at 12 months had 1·9 times (*β* = 0·63, *P* = 0·03) more chance of recovery at 24 months of age. And, children who were stunted at 18 months of age even had higher odds (adjusted OR = 3·01, *β* = 1·10, *P* < 0·001) of recovery than children who were stunted at 6 months. Additionally, mother’s height (*β* = 0·59, *P* = 0·04) and household income (*β* = 0·02, *P* < 0·05) showed statistically significant associations with the outcome.

**Conclusions:**

The study provided evidence for the role of timing of stunting on the recovery from the phenomenon. This novel finding indicates that the programmes to promote linear growth should be directed at the earliest possible timepoints in the course of life.

Stunting or linear growth faltering, an indicator of chronic nutrition deprivation, is known to be an outcome of socioeconomic and biological insults attained before and after the birth of a child^(1)^. Globally, nearly a quarter of the children are stunted, and the rate of reduction is slower, resulting in poor economic growth, particularly in a resource-limited setting^(2)^. Studies conducted in different parts of the world elucidated the role of stunting in elevating the risk of childhood mortality, as well as increasing the susceptibility to infection and thus hampering early childhood growth and developments^(3,4)^. Multiple studies have examined the contribution of different dietary and non-dietary factors in linear growth retardation, and the water, sanitation and hygiene (WASH)-related behaviours were also in the list^(5,6)^. As a result of ingesting large quantities of faecal bacteria, children living in poor WASH conditions often suffer from environmental enteric dysfunction (EED)^(7,8)^. EED is a subclinical disorder of the small intestine characterised by villous atrophy, crypt hyperplasia, increased intestinal permeability and infiltration of inflammatory cells^(7,9)^. EED causes nutrient malabsorption and may result in chronic growth retardation and eventually stunting^(8)^.

After birth, a child’s length-for-age *z*-score (LAZ) starts from close to the standard but falters dramatically in the later part of life^(10)^. This trajectory shows similar patterns across different regions of the World – Asia, Africa and Latin America – and appears to remain unchanged for decades^(10)^. However, there is evidence about the recovery from this linear growth retardation over time^(11,12)^. Compensating a fraction of the early childhood growth retardation is possible if the developmental maturation is delayed and the growth period is prolonged^(13)^. Such maturational delays can be seen among children <2 years of age living in developing countries^(13)^. In addition, available reports also stated that the predictors of stunting are not the same for recovery from this condition^(14)^. Epidemiological research following different designs was employed to clarify the role of WASH practice on the development or prevention or moderation of stunting^(15–17)^. But none of those studies identified the role of WASH in recovery from stunting. Along with determining its predictive value for growth faltering and stunting, the role of WASH in recovery from stunting should also be explored. Hence, the primary objective of the current analysis was to measure the role of WASH practices in recovery from stunting using a multi-country birth cohort data. Additionally, we assessed the role of timing of stunting on recovery from this phenomenon.

## Methodology

### Conceptual framework

**Fig. 1:**
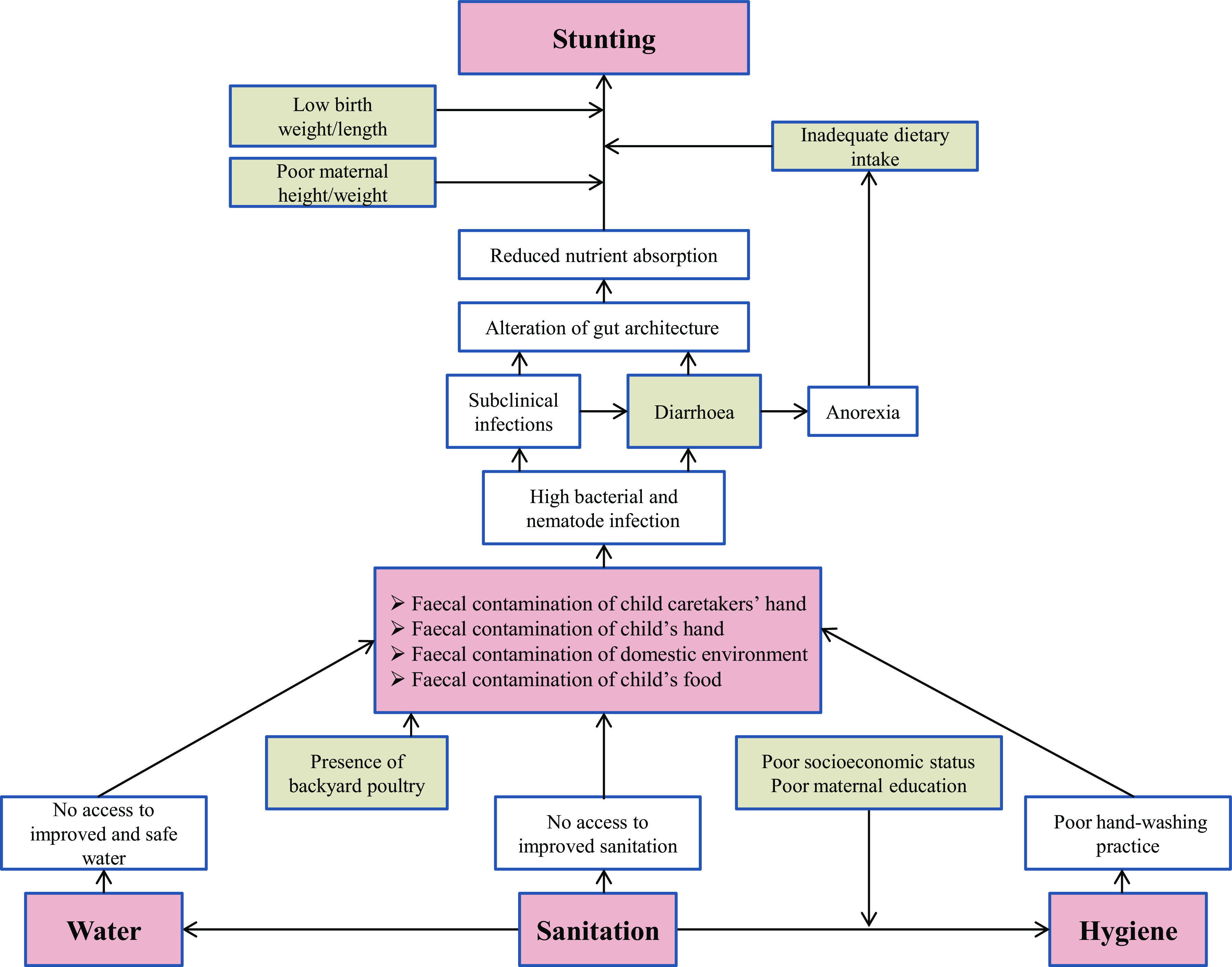
Conceptual framework depicting the water, sanitation and hygiene (WASH)–stunting causal pathway

Exposure to poor WASH environment induces diarrheal diseases and other subclinical infections^(7)^. This causal pathway is further modified by poverty and poor maternal education^(18)^. The other covariates that are known to play a crucial role in WASH–stunting hypothetical framework are low birth weight and height, inadequate energy from protein, and low maternal height and weight^(10)^. We developed a conceptual framework (Fig. 1) based on the abovementioned context, which was used for variable selection and data analysis. Underlying causes of stunting other than those diagrammed here are either primary causes or effect modifiers in the proposed pathway.

### Study setting

Data for this specific analysis was collected from the MAL-ED (Etiology, Risk Factors, and Interactions of Enteric Infections and Malnutrition and the Consequences for Child Health) birth cohort study. The MAL-ED study was conducted from 2 November 2009 to 28 February 2014 at eight different sites across three continents. A total of 2145 children from Dhaka, Bangladesh (BG), Vellore, India (IN), Bhaktapur, Nepal (NP), and Naushahro Feroze, Pakistan (PK), in Asia; Fortaleza, Brazil (BR), and Loreto, Peru (PE), in the Americas; and Venda, South Africa (SA), and Haydom, Tanzania (TZ), in Africa were enrolled within 17 d of their birth and followed uniformly up to 24 months of age. Enrolment took place over a 2-year period with the goal of enrolling 200 children per site. The detailed study design is described elsewhere^(19)^.

### Variable selection and data collection

Variables used in the current analysis are: access to improved water (yes/no), access to improved sanitation (yes/no), treat water to make it safe (yes/no), caregiver washes her hands after using the toilet (never/rarely or sometimes/always), caregiver washes her hands before preparing food (never/rarely or sometimes/always), caregiver washes her hands after helping the child to defecate (never/rarely or sometimes/always), mother’s height, weight and educational status, asset index, household income, energy from protein intake, birth LAZ, birth weight-for-age *z*-score, diarrhoea episodes, exclusive breastfeeding days, and minimum dietary diversity (yes *v*. no).

Demographic and socioeconomic status (SES) questionnaires were adopted from the DHS questionnaires, and water and sanitation sources were defined as improved (or not) based on the WHO criteria^(20)^. Data on WASH practice was collected at 6, 12, 18 and 24 months of child’s age. But, the data did not show any significant variations over time (see online supplementary material, Supplemental Fig. 1). Hence, to avoid additional complexity and to ensure temporality, WASH data collected at 6 months of age was used for the current analysis. The household asset index was constructed using household asset data obtained from the SES questionnaire. From these asset-related dichotomous variables, a common factor score for each household was generated using principal components analysis. Trained field workers visited the households twice in a week to collect intensive dietary and morbidity data. Twenty-four-hour food frequency data was collected monthly from 9 to 24 months of age for assessing child’s dietary and energy intake. The 24-h multiple-pass dietary recall approach was used for this purpose^(21)^. A food composition table, which was locally adapted, was used to calculate the amount of energy taken from the documented diet^(22)^. From the nutrient intake group, the amount of ‘energy from protein’ was selected because a multi-country analysis of the same data revealed ‘lower per cent of energy from protein’ to be an important factor contributing to the odds of being in a lower length-for-age category at 24 months^(10)^. Moreover, all the specific food groups (carbohydrate, protein and fat) were highly correlated to each other. Minimum dietary diversity (MDD), a core indicator of infant and young child’s feeding practice, was used to measure the appropriateness of the complementary feeding practice of children^(23)^. Data on MDD was collected on 6th, 7th and 8th months of child’s age. MDD was a binary variable – ‘yes’ was indicated by 1, and ‘no’ by 0. An MDD score was developed by adding the MDD values of 6th, 7th and 8th months. If the total score was ≥2, the child was mentioned as having MDD.

To document the breastfeeding status, the data collector questioned the mother about the child’s food consumption over the past 24 h. If the response was similar to the WHO definition of exclusive breastfeeding (no other food or drink, not even water – except breast milk – including milk expressed, ORS, drops and vitamins, minerals and medicinal syrups), then the child was considered to be exclusively breastfed. Instead of exclusive breastfeeding status (yes *v*. no), exclusive breastfeeding days was used as it counts the specific number of days. Diarrhoea is defined as having ≥3 loose stools in a 24-h period or at least one loose stool with blood reported by the mother^(24)^. A diarrheal episode is defined as being separated from another episode by at least ≥2 diarrhoea-free days^(24)^.

Using a common protocol, trained field workers measured anthropometric indices monthly up to 24 months of age. Measuring boards were used to measure the length to the nearest 0·1 cm, and digital scales were used to measure the weight of the children to the nearest 10 g^(10)^. LAZ and weight-for-age *z*-score for each child was determined using the WHO 2006 Child Growth Standards^(25)^. A child with LAZ <–2 was classified as stunted^(25)^. Enrolment weight and length, which was taken within first week of birth, was used as the surrogate measure for birth anthropometry. Standard wooden height-measuring boards and bathroom scales were used for measuring maternal height and weight.

### Statistical analysis

Children who became stunted at any of the timepoints of 6, 12 or 18 months of age but not found to be stunted at 24 months of age were classified as having recovered from stunting. A total of 626 children became stunted at 6, 12 or 18 months of age; of them, 130 could recover. Children who were stunted on multiple occasions were counted under the first month of onset. Out of these 626, fourteen participants had missing values. After excluding the ID with missing data, a total of 612 children’s data were available for the current analysis. Out of these 612 children, 127 constituted the ‘recovery from stunting’ group, and the rest remained as the non-recovery group. We first described the overall and country-wise household, maternal and nutritional status of the children using mean, standard deviation and percentages. A comparison of LAZ trajectory between recovered and non-recovered children was done using line graphs, and the rate of recovery was reported as a percentage of children who were stunted at 6 months of age but not at 24 months of age and likewise. This multi-country dataset contains clusters of non-independent observational units, namely ‘country’. Measurements within a country might be more similar than measurements from different countries. Moreover, the cluster sizes were also unequal. To adjust this clustering effect, we used generalised linear mixed-effects models (GLMM) where the intercept of the variable ‘country’ was kept as random. This approach allows a robust estimation of variance in the outcome variable within and between the clusters^(26)^. GLMM estimated the probability of recovery from stunting with WASH practice as the exposure of interest, adjusting for all the other possible covariates. We began with the base model (Table 2, model 1) that was built with the fixed effects of WASH variables. Then, keeping the variables of the base model fixed, other covariates were added one after another according to the conceptual framework. Hence, a model was nested in its next model as it contained all the predictor variables used to build the previous model, plus at least one additional variable. This means that variables of a model were a subset of the next model. Model selection was done based on information-theoretic model selection procedures – Akaike Information Criterion (AIC) and Bayesian information criterion (BIC). Information-theoretic methods are equally applicable for both nested and non-nested models and can provide better estimate statistics to quantify the extent of differences between models than other model selection methods^(27,28)^. The model showing the lowest AIC and BIC values was selected as the final model (model 41, Supplemental File 2). Along with the WASH variables, the fully adjusted final model (Table 2, model 2) contains mother’s height, LAZ at birth, gender and income as fixed effects. During exploratory data analysis, we noticed that timing of stunting could modify the odds of recovery from stunting. Therefore, to see the effect of timing of stunting on recovery, we developed the third generalised linear mixed-effects model (model 3, Table 2). We created a variable named ‘timing of stunting’ with three criteria, stunted at 6 months, stunted at 12 months and stunted at 18 months, and added the variable to model 2. We excluded data from Pakistan because quality assurance procedures identified an unexplained bias in a subset of length measurements^(10)^. Data analysis was conducted in R (version 3.5.1), and lme4 package was used for GLMM^(29)^.

**Table 1:** Sociodemographic, birth, maternal characteristics and water, sanitation, and hygiene (WASH) practice status of the participants

		All (*n* 612)			BG (*n* 106)			BR (*n* 8)			IN (*n* 112)			NP (*n* 49)			PE (*n* 100)			SA (*n* 84)			TZ (*n* 153)		
*n* at enrolment			1868			265			233			251			240			303			314			262	
*n* at 24 months			1471			210			165			227			227			194			237			211	
Sociodemographic, birth, maternal characteristics and WASH practice status																									
		*n*		%	*n*		%	*n*		%	*n*		%	*n*		%	*n*		%	*n*		%	*n*		%
Sex	Male	352		57·52	60		56·60	7		87·50	59		52·68	28		57·14	65		65·00	53		63·10	80		52·29
Exclusive breastfeeding	Yes	15		2·45	12		11·32	1		12·50	1		0·89	0		0	1		1	0		0	0		0
Mother’s age	<18 years	36		5·89	0		0	2		25	7		6·31	0		0	15		15	11		13·1	1		0·65
	18–30 years	440		72·01	90		84·91	6		75	99		89·19	39		79·59	61		61	50		59·52	95		62·09
	>30 years	135		22·09	16		15·09	0		0	5		4·5	10		20·41	24		24	23		27·38	57		37·25
Mother’s age at first pregnancy	<18 years	175		28·64	37		34·91	4		50	22		19·82	2		4·08	58		58	25		29·76	27		17·65
Maternal education	None	75		12·27	22		20·75	0		0	14		12·61	5		10·2	1		1	0		0	33		21·57
	1–5 years	147		24·06	46		43·4	1		12·5	26		23·42	13		26·53	26		26	3		3·57	32		20·92
	>5 years	389		63·67	38		35·85	7		87·5	71		63·96	31		63·27	73		73	81		96·43	88		57·52
Improved water	Yes	491		80·23	106		100	8		100	112		100	49		100	92		92	73		86·9	51		33·33
Improved sanitation	Yes	333		54·41	106		100	8		100	51		45·54	49		100	26		26	81		96·43	12		7·84
Treat water to make it safe	Yes	189		30·88	60		56·6	0		0	13		11·61	15		30·61	19		19	8		9·52	74		48·37
Always wash hands after helping the child to defecate		307		50·16	79		74·53	3		37·5	33		29·46	33		67·35	88		88	47		55·95	24		15·69
Always wash hands before preparing food		223		36·44	21		19·81	3		37·5	17		15·18	20		40·82	80		80	51		60·71	31		20·26
Always wash hands after using the toilet		363		59·31	80		75·47	3		37·5	57		50·89	40		81·63	95		95	56		66·67	32		20·92
Presence of backyard poultry		246		40·20	5		4·72	0		0	13		11·61	18		36·73	43		43	33		39·29	134		87·58
		Mean		sd	Mean		sd	Mean		sd	Mean		sd	Mean		sd	Mean		sd	Mean		sd	Mean		sd
Birth weight (kg)		3·01		0·50	2·64		0·4	3·37		0·37	2·84		0·48	2·88		0·44	2·99		0·42	3·21		0·42	3·31		0·47
Birth length (cm)		48·30		2·09	47·51		2·02	49·03		2·55	48·52		2·16	48·59		1·89	48·04		1·78	49·07		1·76	48·32		2·3
Mother’s height (cm)		151·46		6·56	147·61		4·87	154·1		7·85	150·09		5·03	146·75		4·54	149·15		5·53	156·86		6·18	155·03		6·03
Monthly family income (in USD)		125·97		151·66	130·17		75·26	351·71		75·62	82·46		40·22	157·54		105·74	144·35		84·25	290·21		296·01	30·82		49·44

BG, Bangladesh; BR, Brazil; IN, India; NP, Nepal; PE, Peru; SA, South Africa; TZ, Tanzania; All, all countries combined.

**Table 2:** Parameter estimates for the fixed effects of water, sanitation and hygiene (WASH) and timing of stunting on recovery from stunting from the fully adjusted model

Variables	Model 1 (WASH variables)			Model 2 (WASH variables and other covariates)			Model 3 (WASH variables, timing and other covariates)		
	Regression coefficient		95 % CI	Regression coefficient		95 % CI	Regression coefficient		95 % CI
Access to improved water (yes)	0·54		−0·18, 1·26	0·53		−0·22, 1·27	0·53		−0·24, 1·29
Access to improved sanitation (yes)	0·59*		0·00, 1·18	0·60		0·00, 1·20	0·59		−0·03, 1·20
Treat water to make it safe (yes)	0·02		−0·47, 0·52	0·06		−0·45, 0·57	0·01		−0·50, 0·53
Caregiver washes her hands after helping the child to defecate (Ref. = never)									
Rarely or sometimes	−0·20		−1·01, 0·62	−0·09		−0·92, 0·75	−0·11		−0·95, 0·74
Always	−0·43		−1·29, 0·42	−0·39		−1·27, 0·49	−0·37		−1·25, 0·51
Caregiver washes her hands before preparing food (Ref. = never)									
Rarely or sometimes	0·51		−0·16, 1·18	0·37		−0·32, 1·06	0·44		−0·26, 1·13
Always	0·43		−0·32, 1·17	0·30		−0·47, 1·07	0·36		−0·42, 1·13
Caregiver washes her hands after using the toilet (Ref. = never)									
Rarely or sometimes	0·86		−0·17, 1·90	0·79		−0·26, 1·84	0·77		−0·28, 1·82
Always	1·02*		0·01, 2·04	0·92		−0·12, 1·95	0·81		−0·23, 1·85
Mother’s height				0·61**		0·23, 0·99	0·59**		0·20, 0·97
LAZ at birth				0·29*		0·06, 0·51	0·12		−0·12, 0·36
Sex (male)				−0·46*		−0·88, −0·03	−0·36		−0·79, 0·08
Household income				0·01		0·00, 0·03	0·02*		0·00, 0·03
Timing of stunting (Ref. = stunted at 6 months)									
Stunted at 12 months							0·63*		0·07, 1·20
Stunted at 18 months							1·10***		0·52, 1·69
AIC		622·8			602·6			592·3	
BIC		671·4			668·8			667·4	
Log likelihood		−300·4			−286·3			−279·1	

AIC, Akaike Information Criterion; BIC, Bayesian information criterion.

*P < 0·05, **P < 0·01, ***P < 0·001.

## Results

Table 1 describes the sociodemographic, birth, maternal characteristics and WASH practice status of the participants. The overall male-to-female ratio was 1:0·7. Overall, 64 % of mothers had >5 years of education, the rate of which was the lowest in Bangladesh and the highest in South Africa. Nearly 80 % of the households had access to improved water, and 54 % had access to improved sanitation. However, only 31 % of the households treated water to make it safe to drink. More than 50 % of caregivers always washed their hands after helping the child to defecate and also after using toilet. But, only 36 % washed their hands before preparing foods.

Brazilian children had the highest mean birth weight, and South African children had the highest mean birth length, whereas children born in Bangladesh had the lowest birth weight and birth height. In terms of maternal height, the lowest was observed in Nepal, and South African mothers had the highest. Mean monthly household income was 126 USD, which ranges from 31 USD (Tanzania) to 352 USD (Brazil). In a nutshell, factors that were analysed under the current study exhibited substantial heterogeneity across the countries.

**Fig. 2:**
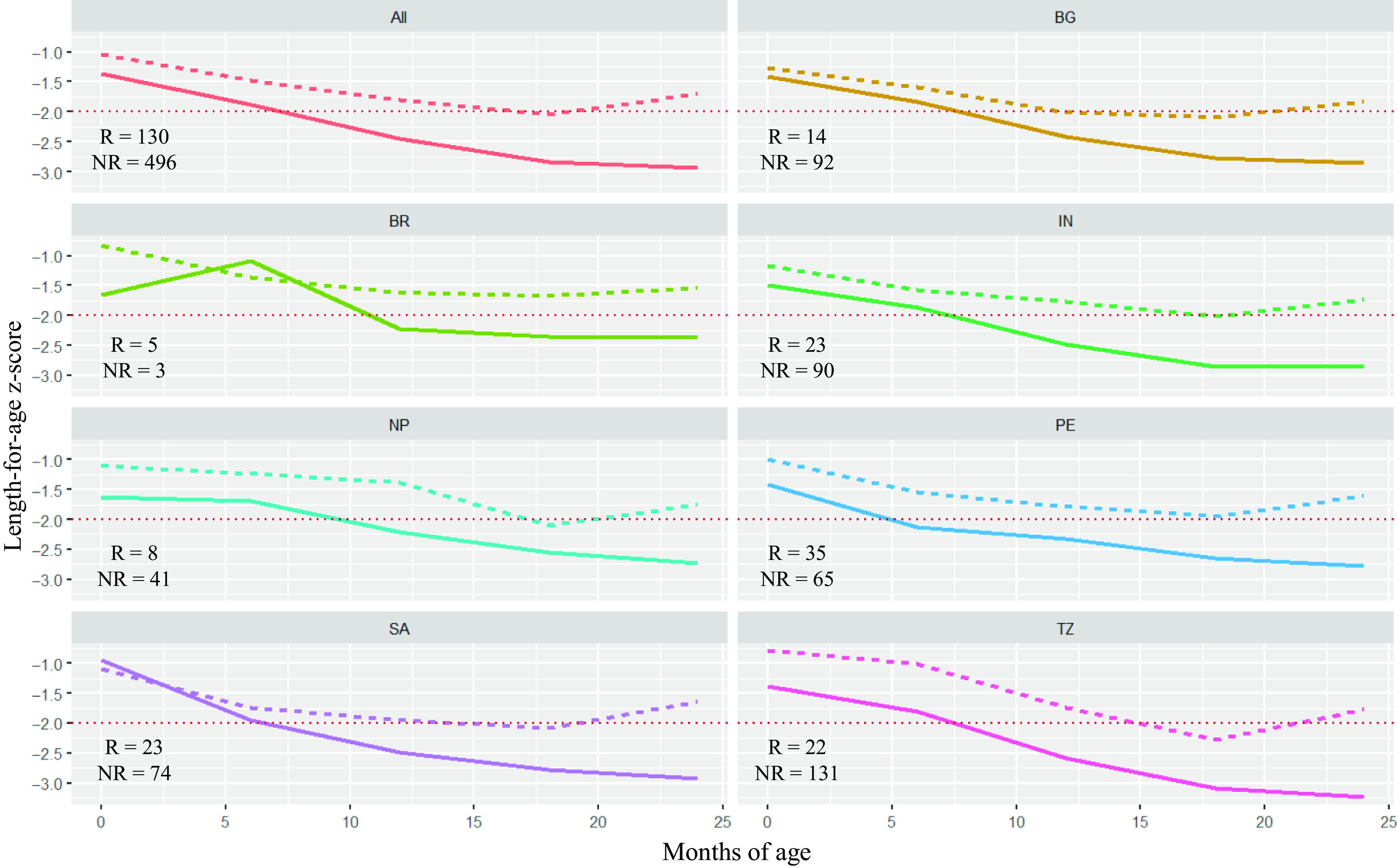
Comparison of length-for-age *z*-score (LAZ) trajectory between recovered and non-recovered children from birth to 24 months of age (BG (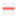): Bangladesh; BR (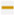): Brazil; IN, (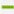): India; NP (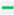): Nepal; PE (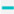): Peru; SA (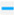): South Africa; TZ (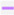): Tanzania; All (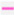): all countries combined). The dotted lines indicate the LAZ score at −2 level. Numbers presented in each graph represent the sample size of recovered (R) and non-recovered (NR) groups. Recovered: 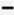, No; 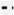, Yes

Figure 2 shows the LAZ trajectory of recovered and non-recovered children from birth to 24 months of age. At birth, the mean LAZ in the recovery group was −1·04 and in the non-recovery group was −1·37. Similar trends can be seen in most of the countries where children who could recover from stunting had a better LAZ at birth than their counterparts, except South Africa.

**Fig. 3:**
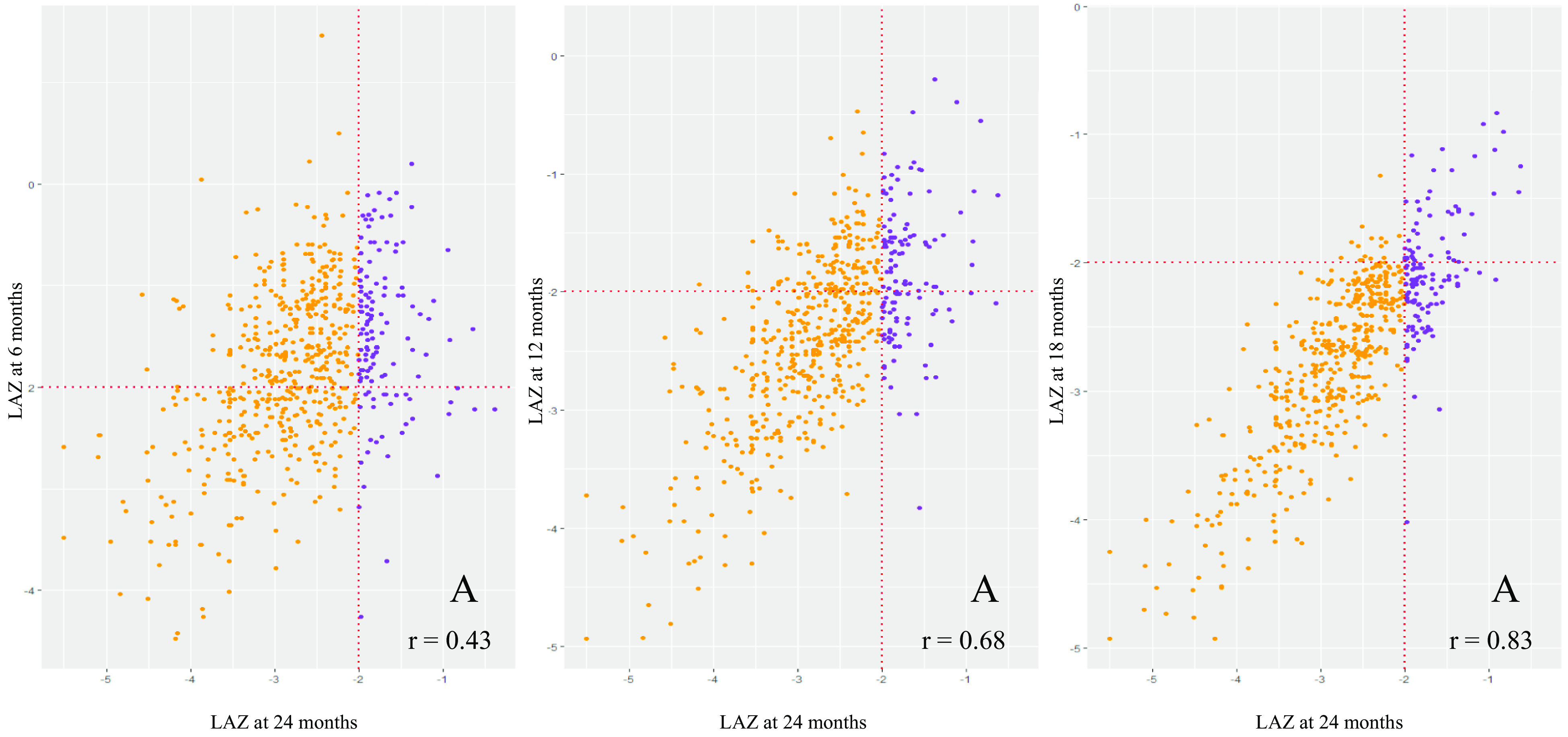
Multi-panel scatter plots presenting the rate and trend of recovery from stunting. Dotted lines indicate the length-for-age *z*-score (LAZ) at −2 level, and the fourth quadrant of each of the plot (marked ‘A’) represents children who were stunted at 6, 12 and 18 months of age, but not at 24 months of age. ‘*r*’ represents the correlation coefficient between LAZ at 24 months and LAZ scores at 6, 12 and 18 months, respectively. Reversed: 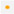, No; 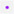, Yes

Figure 3 shows the rates and trends of recovery from stunting and the patterns of correlation between LAZ of children at 6, 12, 18 and 24 months of age. At 6 months of age, 283 children were stunted; of them, only thirty-one (11 %) could recover at 24 months. 434 and 586 children were stunted at 12 and 18 months; of them, fifty-one (12 %) and eighty (14 %) could recover, respectively. This finding indicates that though the overall rate of recovery was low, the chances of recovery were more for children who were stunted at later months than children who were stunted at earlier months of life. The trend of recovery shown in the figure is biologically plausible as LAZ at 18 months were more correlated to LAZ at 24 months (correlation coefficient, *r* 0·83) than LAZ at 6 (*r* 0·43) and 12 months (*r* 0·68).

Table 2 presents the parameter estimates for the fixed effects of WASH and timing of stunting on recovery from stunting from the fully adjusted model after controlling for mother’s height, child’s LAZ at birth, sex and household income. Model 2 indicates that recovery from stunting did not show any statistically significant association with any of the WASH variables (*P* ≥ 0·05). Model 3 shows that timing of stunting had a strong and statistically significant association with recovery from stunting. In comparison with children who were stunted at 6 months, children who were stunted at 12 months had 1·9 times (coefficient *β* = 0·63, *P* < 0·05) more chances of recovery at 24 months of age. And, children who were stunted at 18 months of age even had higher odds (adjusted OR = 3·01, *β* = 1·10, *P* < 0·001) of recovery than children who were stunted at 6 months.

Model 2 reports that recovery from stunting had statistically significant positive association with mother’s height (*P* = 0·002) and LAZ at birth (*P* = 0·01), and negative association with male sex (*P* = 0·03). However, after adjusting for timing of stunting, model 3 reports that the association of LAZ at birth and male sex proved to be statistically insignificant. In addition, household income showed a statistically significant marginal association (*β* = 0·02, *P* < 0·05) with the outcome. Among the three models reported here, model 3 explains the maximum variance (lowest AIC and BIC values, Table 2).

## Discussion

We found that not WASH practice but timing of stunting, household income and maternal height had statistically significant association with recovery from stunting. Importantly, our analysis revealed that children who were stunted at an older age had higher chances of backtracking to normal in 24 months of age. But, there remains a paucity of knowledge explaining such trends of growth. However, the ‘canalisation of growth’ theory states that, under favourable conditions, each individual’s growth trajectory will follow a genetically determined path^(30)^. Any growth-hindering factor can deflect the child away from that course, but after the reappearance of favourable conditions, the child could return to the expected growth channel^(31)^. Prior reports asserted that at the end of a growth retardation period, the affected child grows more rapidly than usual and catches up his or her original growth trajectory^(31)^. To ensure catch-up growth, protective support from the immune system is required^(32,33)^. Multiple studies have stated that the rate at which individuals acquired immune activation gained efficiency with increased age^(34,35)^. This can be a possible explanation of our findings regarding the role of timing of stunting on the reversal phenomenon of stunting in this cohort of children.

This explanation is further supported by the advent of household income as one of the factors contributing to the outcome in our analysis. Evidence from Bangladesh reveals that total household expenditures and household income are significantly associated with household dietary diversity score^(36,37)^. Several studies have also identified dietary diversity as a useful indicator of micronutrient intake among infants and young children^(38,39)^. Micronutrients such as zinc, selenium, iron, copper, vitamins A, C, E and B_6_ and folic acid support the immune system by strengthening epithelial barriers as well as cellular and humoral immune responses^(40,41)^. Inadequate intake of these vitamins and trace elements may cause immune suppression and could direct a child towards infection and eventually malnutrition. From this discussion, we can hypothesise that children from economically solvent households took diversified foods, which in turn helped them boost their immune system and recover from stunting. But this statement demands further exploration.

Our study reported that children with a higher LAZ at birth and with a better maternal stature had better chances of recovery from stunting than their counterparts. Studies done in a similar setting have reported the role of prenatal factors in the development of stunting^(10,42)^. What our study adds to the existing knowledge is that higher LAZ at birth and better maternal height also increase the chances of child’s recovery from stunting. However, the association between birth LAZ and chances of recovery from stunting became statistically insignificant when ‘timing of stunting’ was added to the final model.

The primary objective of the current study was to measure the role of WASH practices on recovery from stunting. After adjusting for the confounders, our study could not find any significant effect of WASH practice on recovery from stunting. Though we could not find any descriptive studies describing this relation, a recently conducted cluster-randomised trial has found that there is no effect of water, sanitation and hand-washing on the linear growth of children^(15)^. Similar reports are available from at least three other recently completed trials^(16)^. Consistent with those reports, the contribution of WASH variables became insignificant in our final model after adjusting for other covariates. But this finding should not undermine the role of WASH on undernutrition in young children. Data pertaining to the relationship between WASH and linear growth retardation are paradoxical, and it is also evident that children living in cleaner households had a lower prevalence of stunting than those from contaminated households^(17)^. It indicates that the pathways linking poor WASH practice to stunting are complex. Hence, further evaluation is required to determine the exact contribution of WASH practices on linear growth impairment. Based on the findings of the current study, at best we can hypothesise that only the provision of adequate WASH facilities and good hygiene practices would not eliminate stunting in resource-poor settings such as Bangladesh.

Our study used the data of a multi-county birth cohort study. Hence, we expect our results to be generalisable to the under-two children living in different parts of the world. This is the strength of the current analysis. However, the WASH practices were determined based on the self-reported data from the mothers/caregivers of the children. Hence, chances of reporting bias could not be averted. Moreover, gestational age at delivery was not recorded. This might have an implication in assessing the length-for-age of children. These are the limitations of our analysis that must be reported.

In conclusion, our findings provide a stringent evidence of the role of WASH practices on childhood linear growth impairment. There was no effect of WASH behaviours on recovery from stunting in this cohort of children. However, the timing of stunting had a statistically significant relationship with recovery from stunted growth at 24 months of age. This novel finding indicates that programmes to prevent linear growth impairment should be directed at the earliest possible timepoints in the early course of life.

## Supplementary material

For supplementary material accompanying this paper visit https://doi.org/10.1017/S136898002000004X. click here to view supplementary material
