# Supplementary material for: Not water, sanitation and hygiene practice, but timing of stunting is associated with recovery from stunting at 24 months: results from a multi-country birth cohort study
Source: Public Health Nutr. 2020 May 14;24(6):1428–37. doi: 10.1017/S136898002000004X (PMC8025093; doi:10.1017/S136898002000004X)
Supplement: Supplementary file 1 [file S136898002000004Xsup.zip › S136898002000004Xsup002.pdf]

Model	Variables	AIC	BIC
1 (BASE)	Recovery of stunting ~ improved water + improved sanitation + treat water to make it safe + wash hand after helpin the child to defecate + wash hand before cooking food + caregiver washes her hands after cleaning the child’s bottom	622.8	671.4
2	Base + household has chicken or ducks	624.7	677.7
3	Base + Maternal education	624.4	677.4
4	Base + asset	623.6	676.6
5	Base + income	619.7	672.7
6	Base + energy from protein	623.8	676.8
7	Base + gender	621.9	674.9
**8**	**Base + Mothers' height**	**611.4**	**664.4**
9	Base + birth LAZ score	615.2	668.2
10	Base + birth WAZ score	620.3	673.3
11	Base + Mother's weight	613.2	666.2
12	Base + exclusive breastfeeding days	622.7	675.7
13	Base + diarrhea episodes	624.7	677.7
14	Base + MDD	623.4	676.4
15	Base + Mothers' height + household has chicken or ducks	613.4	670.8
16	Base + Mothers' height + Maternal education	613.4	670.8
17	Base + Mothers' height + asset	612.8	670.2
18	Base + Mothers' height + income	609.5	667
19	Base + Mothers' height + energy from protein	612.7	670.1
20	Base + Mothers' height + gender	609	666.4
**21**	**Base + Mothers' height + birth LAZ score**	**607**	**664.5**
22	Base + Mothers' height + birth WAZ score	611.1	668.5
23	Base + Mothers' height + Mother's weight	608.9	666.3
24	Base + Mothers' height + Exclusive breastfeeding days	611.7	669.1
25	Base + Mothers' height + Diarrhea episodes	612.7	670.1
26	Base + Mothers' height + MDD	613	670.4
27	Base + Mothers' height + birth LAZ score + household has chicken or ducks	609	670.9
28	Base + Mothers' height + birth LAZ score + Maternal education	609	670.9
29	Base + Mothers' height + birth LAZ score + asset	608.4	670.2
30	Base + Mothers' height + birth LAZ score + income	605	666.9
31	Base + Mothers' height + birth LAZ score + energy from protein	608.3	670.2
**32**	**Base + Mothers' height + birth LAZ score + gender**	**604.5**	**666.3**
33	Base + Mothers' height + birth LAZ score + birth WAZ score	608.9	670.7
34	Base + Mothers' height + birth LAZ score + Mother's weight	605.8	667.6
35	Base + Mothers' height + birth LAZ score + Exclusive breastfeeding days	607.1	668.9
36	Base + Mothers' height + birth LAZ score + Diarrhea episodes	608.4	670.3
37	Base + Mothers' height + birth LAZ score + MDD	608.5	670.4
38	Base + Mothers' height + birth LAZ score + gender + household has chicken or ducks	606.4	672.7
39	Base + Mothers' height + birth LAZ score + gender + Maternal education	606.5	672.7
40	Base + Mothers' height + birth LAZ score + gender + asset	605.8	672.1
**41**	**Base + Mothers' height + birth LAZ score + gender + income**	**602.6**	**668.8**
42	Base + Mothers' height + birth LAZ score + gender + energy from protein	605.6	671.8
43	Base + Mothers' height + birth LAZ score + gender + birth WAZ score	606.3	672.6
44	Base + Mothers' height + birth LAZ score + gender+ Mother's weight	603.2	669.5
45	Base + Mothers' height + birth LAZ score + gender+ Exclusive breastfeeding days	604.4	670.6
46	Base + Mothers' height + birth LAZ score + gender+ Diarrhea episodes	605.6	671.9
47	Base + Mothers' height + birth LAZ score + gender+ MDD	606	672.3
48	Base + Mothers' height + birth LAZ score + gender + income + household has chicken or ducks	604.6	675.2
49	Base + Mothers' height + birth LAZ score + gender + income + asset	604.4	675.1
50	Base + Mothers' height + birth LAZ score + gender + income + Maternal education	604.6	675.2
51	Base + Mothers' height + birth LAZ score + gender + income + energy from protein	603.9	674.6
52	Base + Mothers' height + birth LAZ score + gender + income + birth WAZ score	604.4	675
53	Base + Mothers' height + birth LAZ score + gender + income + Mother's weight	602.6	673.3
**54**	**Base + Mothers' height + birth LAZ score + gender + income + Exclusive breastfeeding days**	**602.5**	**673.2**
55	Base + Mothers' height + birth LAZ score + gender + income + Diarrhea episodes	603.5	674.2

56	Base + Mothers' height + birth LAZ score + gender + income + MDD	604.2	674.9
57	Base + Mothers' height + birth LAZ score + gender + income + Exclusive breastfeeding days + household has chicken or ducks	604.4	679.5
58	Base + Mothers' height + birth LAZ score + gender + income + Exclusive breastfeeding days + asset	604.4	679.5
59	Base + Mothers' height + birth LAZ score + gender + income + Exclusive breastfeeding days + Maternal education	604.4	679.5
60	Base + Mothers' height + birth LAZ score + gender + income + Exclusive breastfeeding days + energy from protein	603.9	679
61	Base + Mothers' height + birth LAZ score + gender + income + Exclusive breastfeeding days + birth WAZ score	604.3	679.4
**62**	**Base + Mothers' height + birth LAZ score + gender + income + Exclusive breastfeeding days + Mother's weight**	**602.8**	**677.8**
63	Base + Mothers' height + birth LAZ score + gender + income + Exclusive breastfeeding days + Diarrhea episodes	603.6	678.7
64	Base + Mothers' height + birth LAZ score + gender + income + Exclusive breastfeeding days + MDD	604.2	679.2
65	Base + Mothers' height + birth LAZ score + gender + income + Exclusive breastfeeding days + Mother's weight+ household has chicken or ducks	604.7	684.2
66	Base + Mothers' height + birth LAZ score + gender + income + Exclusive breastfeeding days + Mother's weight+ asset	604.7	684.2
67	Base + Mothers' height + birth LAZ score + gender + income + Exclusive breastfeeding days + Mother's weight+ Maternal education	604.7	684.2
68	Base + Mothers' height + birth LAZ score + gender + income + Exclusive breastfeeding days + Mother's weight+ energy from protein	604.2	683.7
69	Base + Mothers' height + birth LAZ score + gender + income + Exclusive breastfeeding days + Mother's weight+ birth WAZ score	604.3	683.8
**70**	**Base + Mothers' height + birth LAZ score + gender + income + Exclusive breastfeeding days + Mother's weight+ Diarrhea episodes**	**604**	**683.5**
71	Base + Mothers' height + birth LAZ score + gender + income + Exclusive breastfeeding days + Mother's weight+ MDD	604.4	683.9
72	Base + Mothers' height + birth LAZ score + gender + income + Exclusive breastfeeding days + Mother's weight+ Diarrhea episodes+ household has chicken or ducks	605.9	689.8
73	Base + Mothers' height + birth LAZ score + gender + income + Exclusive breastfeeding days + Mother's weight+ Diarrhea episodes+ asset	605.9	689.9
74	Base + Mothers' height + birth LAZ score + gender + income + Exclusive breastfeeding days + Mother's weight+ Diarrhea episodes+ Maternal education	605.9	689.8
**75**	**Base + Mothers' height + birth LAZ score + gender + income + Exclusive breastfeeding days + Mother's weight+ Diarrhea episodes+ energy from protein**	**605.5**	**689.4**
76	Base + Mothers' height + birth LAZ score + gender + income + Exclusive breastfeeding days + Mother's weight+ Diarrhea episodes+ birth WAZ score	605.5	689.4
77	Base + Mothers' height + birth LAZ score + gender + income + Exclusive breastfeeding days + Mother's weight+ Diarrhea episodes+ MDD	605.6	689.5
78	Base + Mothers' height + birth LAZ score + gender + income + Exclusive breastfeeding days + Mother's weight+ Diarrhea episodes+ energy from protein+ household has chicken or ducks	607.4	695.8
79	Base + Mothers' height + birth LAZ score + gender + income + Exclusive breastfeeding days + Mother's weight+ Diarrhea episodes+ energy from protein+ asset	607.5	695.8
80	Base + Mothers' height + birth LAZ score + gender + income + Exclusive breastfeeding days + Mother's weight+ Diarrhea episodes+ energy from protein+Maternal education	607.4	695.7
**81**	**Base + Mothers' height + birth LAZ score + gender + income + Exclusive breastfeeding days + Mother's weight+ Diarrhea episodes+ energy from protein+ birth WAZ score**	**607**	**695.4**
82	Base + Mothers' height + birth LAZ score + gender + income + Exclusive breastfeeding days + Mother's weight+ Diarrhea episodes+ energy from protein+MDD	607.2	695.5
83	Base + Mothers' height + birth LAZ score + gender + income + Exclusive breastfeeding days + Mother's weight+ Diarrhea episodes+ energy from protein+ birth WAZ score+ household has chicken or ducks	609	701.8
84	Base + Mothers' height + birth LAZ score + gender + income + Exclusive breastfeeding days + Mother's weight+ Diarrhea episodes+ energy from protein+ birth WAZ score+ asset	609	701.8
85	Base + Mothers' height + birth LAZ score + gender + income + Exclusive breastfeeding days + Mother's weight+ Diarrhea episodes+ energy from protein+ birth WAZ score+ Maternal education	609	701.7

**86**	**Base + Mothers' height + birth LAZ score + gender + income + Exclusive breastfeeding days + Mother's weight+ Diarrhea episodes+ energy from protein+ birth WAZ score+ MDD**	**608.8**	**701.5**
87	Base + Mothers' height + birth LAZ score + gender + income + Exclusive breastfeeding days + Mother's weight+ Diarrhea episodes+ energy from protein+ birth WAZ score+ MDD+household has chicken or	610.7	707.9
88	Base + Mothers' height + birth LAZ score + gender + income + Exclusive breastfeeding days + Mother's weight+ Diarrhea episodes+ energy from protein+ birth WAZ score+ MDD+asset	610.7	707.9
89	Base + Mothers' height + birth LAZ score + gender + income + Exclusive breastfeeding days + Mother's weight+ Diarrhea episodes+ energy from protein+ birth WAZ score+ MDD+Maternal education	610.7	707.9
