# Supplementary figures and images for: Not water, sanitation and hygiene practice, but timing of stunting is associated with recovery from stunting at 24 months: results from a multi-country birth cohort study
Source: Public Health Nutr. 2020 May 14;24(6):1428–37. doi: 10.1017/S136898002000004X (PMC8025093; doi:10.1017/S136898002000004X)

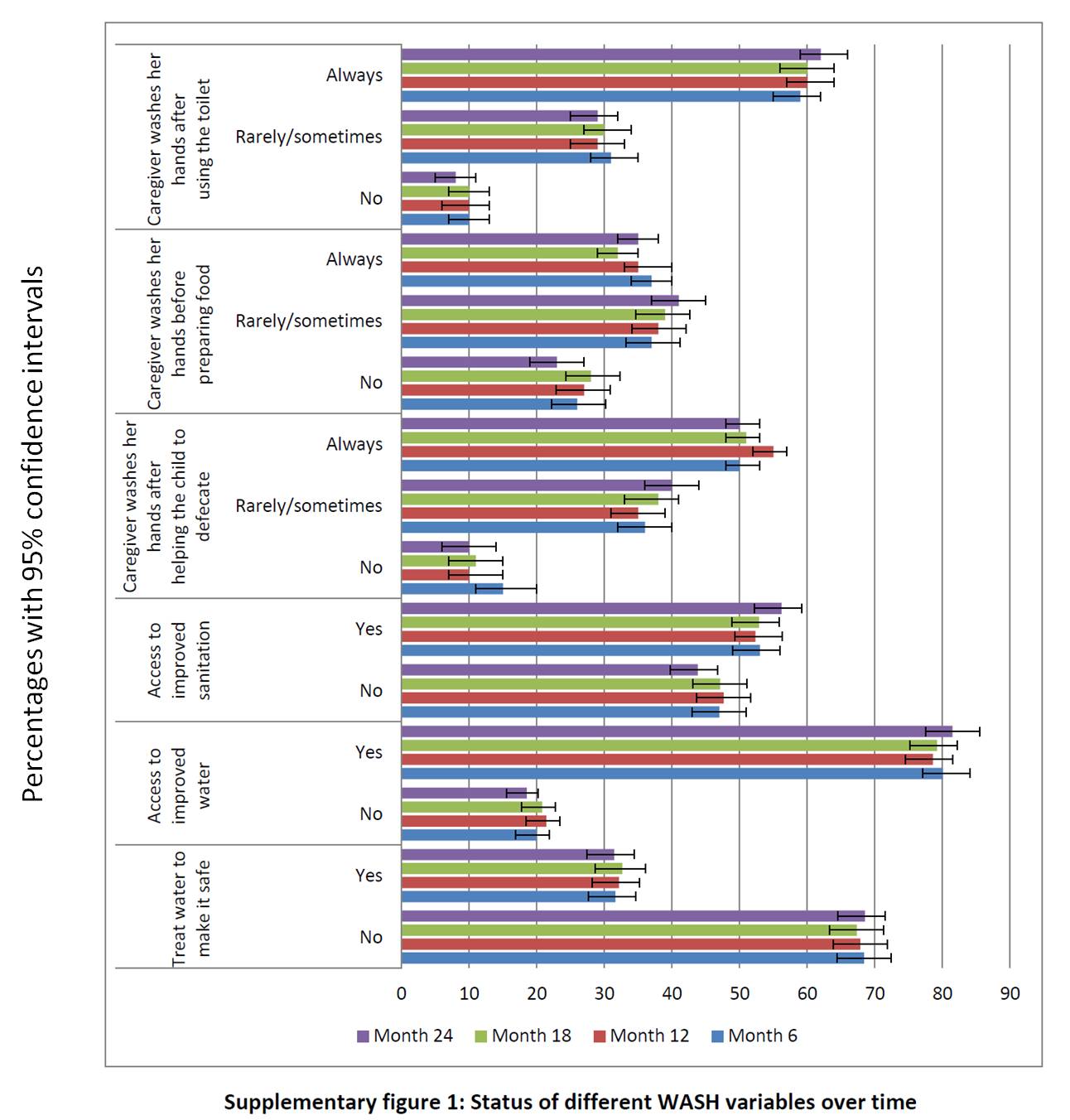

Supplement: Supplementary file 1 [file S136898002000004Xsup.zip › S136898002000004Xsup001.jpg]
